# Morphological variability may limit single-cell specificity to electric field stimulation

**DOI:** 10.3389/fnsyn.2025.1621352

**Published:** 2025-08-05

**Authors:** Daniel Trotter, Aref Pariz, Axel Hutt, Jérémie Lefebvre

**Affiliations:** ^1^Department of Physics, University of Ottawa, Ottawa, ON, Canada; ^2^Krembil Brain Institute, University Health Network, Toronto, ON, Canada; ^3^Department of Biology, University of Ottawa, Ottawa, ON, Canada; ^4^Team MIMESIS, INRIA, UMR7357 CNRS, ICube Research Department, Strasbourg, France; ^5^Department of Mathematics, University of Toronto, Toronto, ON, Canada; ^6^Department of Cellular and Molecular Medicine, University of Ottawa, Ottawa, ON, Canada

**Keywords:** non-invasive brain stimulation (NIBS), transcranial electric stimulation, computational modeling, single neuron model, uniform electric field

## Abstract

**Introduction:**

Non-invasive brain stimulation techniques, widely used to manipulate neural excitability and behavior, are well studied at the meso- and macroscopic scales. However, less is known about their specificity at the level of individual cells.

**Methods:**

Models based on real pyramidal and parvalbumin neuron morphologies created by the Allen Institute for Brain Science were characterized using metrics we devised to quantify various aspects of cellular morphology, ranging from whole cell attributes to net compartment length, branching, diameter and orientation. The models were simulated to quantify the single-cell variability and evoked response susceptibility to uniform electric fields.

**Results and discussion:**

No physical traits yielded layer- or cell-type-specific responses passing statistical significance tests. While uniform electric fields reliably modulated somatic, dendritic and axonal compartments, and subtype-specific responses were observed, specificity was blurred by the variability in cellular morphology. These null results suggest morphology alone may not account for the reported subtype specificity to electric field stimulation, and question the extent to which non-invasive techniques can control specific components of neural circuitry.

## Introduction

Non-invasive brain stimulation (NIBS) paradigms are used for a variety of purposes, including attempted treatment of several psychiatric conditions [e.g. major depressive disorder (Riddle et al., 2020; Haller et al., 2020), schizophrenia (Hasan et al., 2016), bipolar disorder (Piccoli et al., 2022)], and investigating neural oscillation properties and controllability (Hui et al., 2019; Paulus, 2011; Huang et al., 2021). Despite reported clinical efficacy, many questions remain about the mechanisms involved (Liu et al., 2018) as well as how to optimize their use. The high variability in brain response to NIBS protocols represents a major limitation in these efforts. Such variability has been attributed to genetic and neurophysiological factors, but evidence suggests that the brain state during stimulation may further impact the response (Rocchi et al., 2018; Guerra et al., 2020; Lefebvre et al., 2017). Many contributing factors to response variability are immutable (e.g. subject age, skull thickness, etc), but others, such as the placement of the apparatus (e.g. electrodes or coil), are malleable (Guerra et al., 2020; Tremblay et al., 2019). The immense combination of free parameters in these protocols greatly complicates parsing which ones are crucial. This has led to the development of a variety of computational models in efforts to improve the efficacy of experimental approaches and our overall understanding of NIBS (Huang et al., 2021; Mahmud and Vassanelli, 2016).

Despite widespread utility, comparatively little is known about the effects of NIBS at the level of single neurons (Liu et al., 2018). It is known experimentally, although not widely investigated, that NIBS techniques can modulate the activity of individual cells (Krause et al., 2019, 2022; Tremblay et al., 2019). Along with this awareness has come an acute interest in parsing the limits of *specificity* of these techniques: That is, the extent to which neuron traits (e.g. subtype, morphology) govern the ability of a single NIBS protocol to acutely activate or suppress the response of neurons sharing similar characteristics. Preliminary efforts have been made to elucidate a relationship between response variability and neuron morphology via intermediate morphological models (that is, models with a limited set of stereotyped, structural components) with nominal success (Yi et al., 2017; Aspart et al., 2018; Komarov et al., 2019; Aberra et al., 2020; Arnaudon et al., 2023). One such simplified model examined the relationship between many physical traits (e.g., branching, compartment widths and lengths, etc.) of a basic neuron and found that, on a trait-by-trait basis, they alter the strength of electric field (E-field) necessary for the neuron to fire (Yi et al., 2017). Moreover, even for the most simplified case of a straight-cable neuron model (Aspart et al., 2018), changing a single property, such as the length of the cable, was found to influence the response. Collectively this supports the consensus that physical morphology acts as an avenue for layer- and cell-type NIBS specificity.

However, experimental work in alert non-human primates, at field strengths typically applied to humans, has shown transcranial electrical stimulation is capable of recruiting individual neurons and, in some cases, controlling the timing, but not firing rate, of their spikes (Krause et al., 2019). That experimental study found no notable difference in phase locking between putative interneurons and putative pyramidal cells. This result is at odds with what has historically been expected of individual neurons, which are long theorized to have subtype- and layer-specific differences in NIBS response (Radman et al., 2009; Komarov et al., 2019; Huang et al., 2021). In light of this, it may then be purported that other neuron traits (e.g. morphology) that vary widely across neuron populations may in fact limit layer- and cell-type specificity to NIBS paradigms.

Here, we put these results to the test using detailed morphological multi-compartment models from real mouse neurons. The present work employs computational methods to evaluate the effects of NIBS on membrane potential polarization of neurons of different types (PV, PC) populating various cortical layers via the application of a uniform E-field. We specifically examined which, if any, physical characteristics might be involved in generating cell-type and/or layer-specific responses. To do so, we developed metrics quantifying various aspects of cellular morphology, both at the level of whole cells and the compartments they are modeled from, ranging from cell volume to compartment lengths, diameters, number of branches, cellular orientation as well as interneuron myelination (Technical White Paper: Biophysical Modeling—All Active, 2016; Technical White Paper: Biophysical Modeling—Perisomatic, 2017; Call and Bergles, 2021). Using field strengths commensurate with those used in non-invasive experimental settings, such metrics were used as a benchmark for evaluating how polarization varied as a function of cellular morphology, linking single-cell morphological characteristics to response specificity. Axonal, dendritic, and somatic compartments could be modulated reliably through changes in E-field amplitude and orientation, and both subtype and layer-specific responses could be observed. Yet, none of these results could be attributed to morphology: the aforementioned metrics didn't show any statistically significant influence in supporting subtype- and/or layer-specific responses. These null results suggest that the interplay between stimulation parameters and physical neuron characteristics is complex in its direction of neuron excitability and that other biophysical features, such as ion channel density and membrane time constant, might be more relevant. Yet, the abundant variability of these traits both within and between neuron subtypes (Pariz et al., 2023; Moradi Chameh et al., 2021; Cembrowski and Spruston, 2019) may limit layer- and subtype specificity of non-invasive stimulation paradigms. We believe reporting such null results represents a crucial step for the optimization, scope of applicability, and replicability of NIBS studies.

## Results

### Characterizing neuron morphology variability

**Figure 1:**
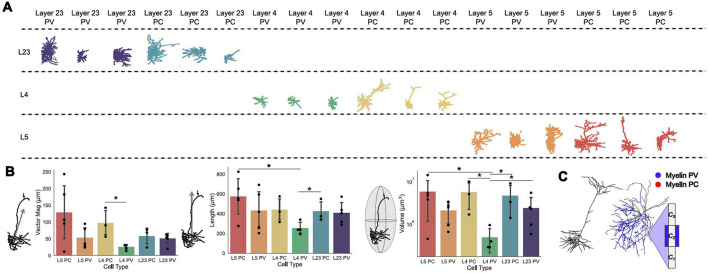
Whole-cell morphological characterization. **(A)** Example morphologies of three neuron models for each type, pyramidal (PC) and parvalbumin (PV), for layers (L) 23, 4, and 5. **(B)** (left) Vector magnitudes of all neuron models split by layer and type; (middle) Length in the z-direction of all neuron models; (right) ellipsoid volume occupied by neuron models. Error bars are standard deviations. All bars are color-matched to the morphologies shown in **(A)**. Significance determined using Mann Whitney U tests; single star represents *p* < 0.05. The numerical values of the mean and standard deviation of each quantity can be found in Supplementary Table 1. **(C)** Example morphologies and schematic for a PC and a PV neuron model with representative myelination on their axons. In **(C)**, *C*_*i*_, *i* = 1, 2, 3 represent the axonal compartment and the blue sheet over *C*_2_ is myelin.

The neuron models created by the Allen Institute are based on the morphologies of pyramidal (PC) and parvalbumin (PV) neurons from layers (L) 23, 4, and 5 of mouse primary visual cortex (Technical White Paper: Biophysical Modeling—All Active, 2016; Technical White Paper: Biophysical Modeling—Perisomatic, 2017) (see Methods). A collection of example morphologies of these models from each layer and subtype are shown in Figure 1A. Seeking to investigate the effects of morphology on the response of such neurons to NIBS is a very high-dimensional problem due to the high variability seen between neurons. We quantified the variability in physical properties between neuronal subtypes at the level of (1) whole cells (i.e., vector magnitude, length, volume, myelin; see Methods); and (2) compartments (i.e., axonal/dendritic compartment lengths, diameters, branches; see Methods) and evaluated the contribution of each on membrane potential polarization from uniform electric fields.

At the whole-cell level, vector magnitude refers to the mean of the square root of the summed squared cartesian coordinates of each compartment. The length refers to the maximum length of the neuron in the z-direction and volume is the maximal elliptical volume it occupies. Separating the mean vector magnitude for each neuron subtype and layer, the PV neurons generally had smaller vector magnitudes, and so were less spatially spread, than their PC counterparts, with L2/3 having the smallest difference between the two subtypes (see Figure 1B, top). In line with this, the PV neurons were on average shorter than their PC counterparts in the same layers, with L2/3 having the minimal difference between subtypes (see Figure 1B, top and middle panels). Similarly to the vector magnitudes and lengths of these cell models, the volumes occupied by neuron subtypes in the same layers were similar, with the exception of L4 where the PV neurons occupied substantially less volume (Figure 1B, bottom). Within subtypes, these macroscopic characterizations were correlated with each other, such that longer neurons tended toward a larger vector magnitude and by extension elliptical volume.

**Figure 2:**
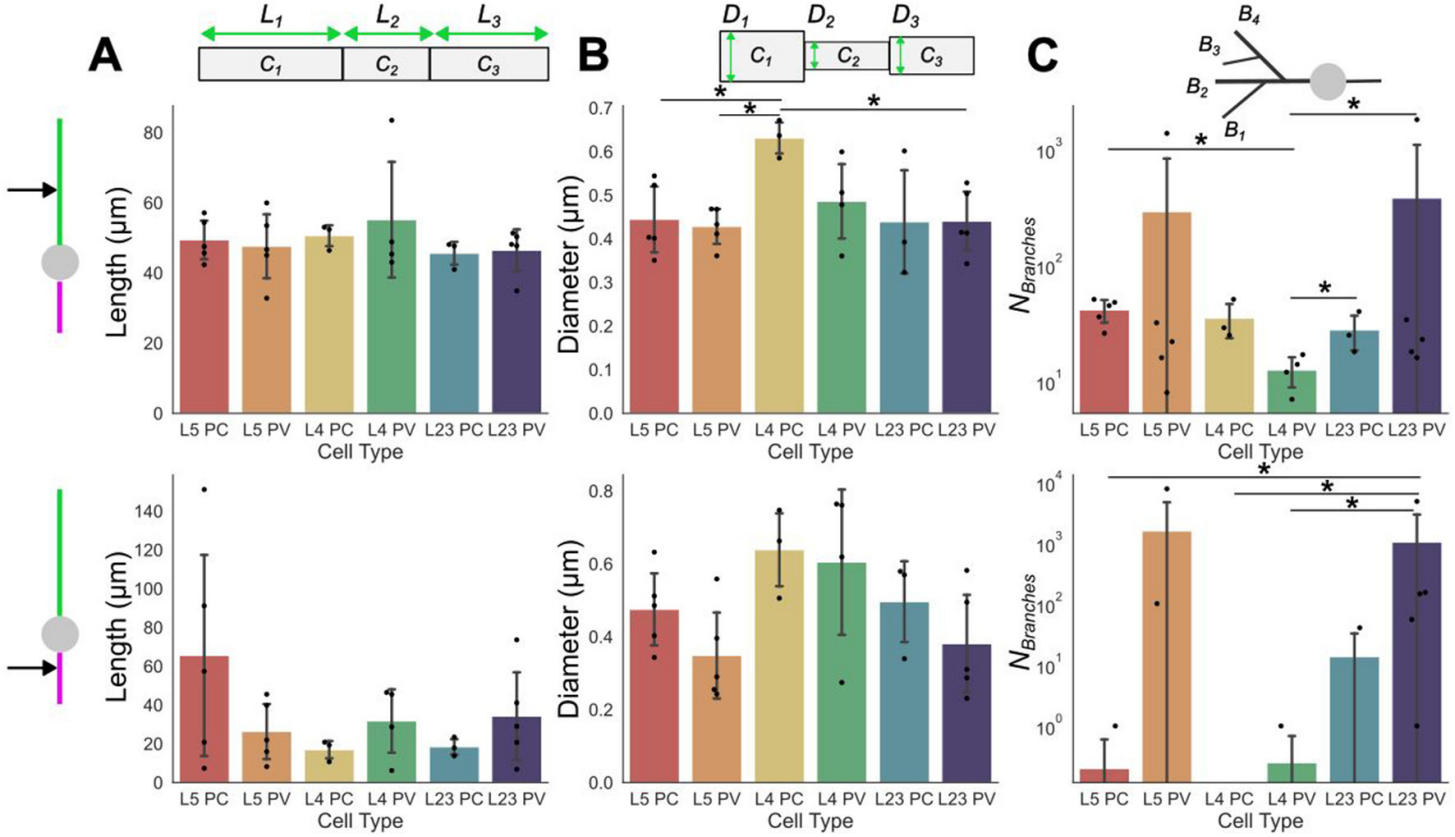
Dendritic and axonal compartment characterizations. Compartmental properties averaged across neuron cell-subtypes of dendrites (top row) and axons (bottom row). Individual points plotted on/above the bars are the compartmental quantity average for individual models; bars are the mean values across models. **(A)** average compartment lengths (*L*_*i*_), **(B)** average compartment diameter (*D*_*i*_). **(C)** average number of branches. Error bars are standard deviations. Significance determined using Mann Whitney U tests; single star represents *p* < 0.05. The numerical values of the mean and standard deviation of each quantity can be found in Supplementary Table 2 (dendtritic compartments) and Supplementary Table 3 (axonal compartments).

In addition to these whole-cell characterizations, compartment-level variability may also be considered (see Methods). These can be separated further into characteristics and organization of dendritic and/or axonal components for each subtype (see Figure 2 top and bottom rows, respectively). This scale of breakdown for the model properties is important as it has been demonstrated that, in isolation, each of these features may affect the field strength required to evoke depolarization (Yi et al., 2017). For these models, the compartment lengths show more variability across the layers and subtypes in the axonal compartments than in the dendritic ones (Figure 2A). Further, the dendritic compartments are on average longer than the axonal compartments, however, there are exceptions such as in L5 PC models where axonal and dendritic compartments were around the same length.

By contrast, diameters are relatively similar between the dendritic and axonal compartments (Figure 2B). The diameters observed between subtypes and layers are also, on average, relatively similar to each other. Branching is, generally, observed to be much more prevalent in the dendritic compartments than the axonal ones (Figure 2C). However, there are exceptions to this, such as in the L5 and L2/3 PV models where the axons are very densely branched. Indeed, the PV models generally have more branching than their PC counterparts in the same layer regardless of whether the branching is considered at an axonal or dendritic level.

### Subtype- and layer-specific membrane potential polarization from uniform electric fields

**Figure 3:**
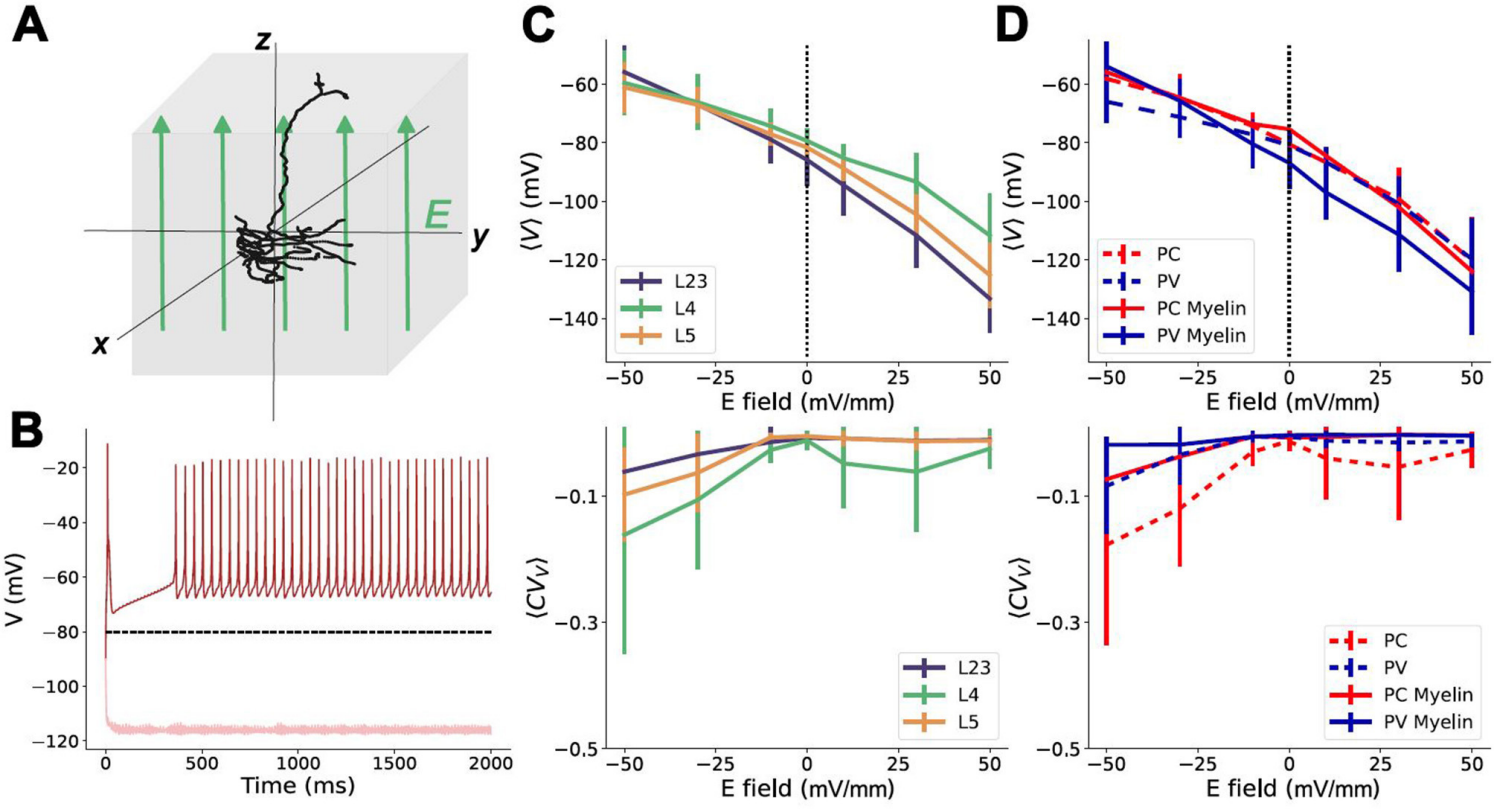
Uniform electric fields impact on somatic membrane potential polarization. **(A)** Schematic of a uniform electric field, *E*, being applied to a neuron model; **(B)** Example single trial responses of the somatic compartment of a PV layer 5 neuron model to a uniform field for *E* = −50 mV/mm (dark red), and *E* = +50 mV/mm (light red). The black dashed line is the average response to noise (*E* = 0 mV/mm) over ten trials. **(C)** Top: Average polarization of the somatic membrane potential (〈*V*〉) across trials pooled by layer. Error bars are the standard deviation measured across trials. Bottom: average coefficient of variation (〈*CV*_*V*_〉) of the membrane potential at different E-field strengths, pooled by layer. Error bars are the standard deviations across trials. **(D)** Top: Average membrane potential across trials pooled by neuron sub-type. Bottom: average 〈*CV*_*V*_〉 of neurons at different E-field strengths pooled by neuron sub-type. Solid lines are myelinated; dashed lines are unmyelinated. Error bars are the standard deviation.

We first examined the mean membrane potential polarization of these models across layers and subtypes, independently of their morphological variability. To do this, we apply a uniform electric field to the neuron models across all its compartments (Figure 3A) (see Methods section). The field sign and strength will influence whether depolarization or hyperpolarization is observed in the neurons' somatic compartments, and influence the neuronal response. We quantified this response through (1) the mean membrane potential polarization (i.e., 〈*V*〉); and (2) membrane potential polarization variability (i.e., 〈*CV*_*V*_〉) across independent trials (see Figure 3B and Methods section). The mean membrane potential polarization quantifies the net effect of field strength on neuronal excitability, while the variability reflects its impact on spiking activity evoked by the applied field. In each trial, neurons were exposed to independent noise realizations of low amplitude, insufficient to cause spontaneous firing.

While not statistically significant (see Methods), differences in mean polarization and polarization variability could be observed between subtypes and across layers. However, pooling the neurons by layer (neglecting subtypes), no significant difference between the mean somatic polarization could be observed. The lowest coefficient of variation of these membrane potentials, 〈*CV*_*V*_〉, is observed for the L2/3 models, with the highest being the L4 models (Figure 3C) indicating increased firing in that layer. By comparison, ignoring layers, and pooling the neuron models based on their subtypes only, there are visible differences between the depolarization responses of PC and PV cells (Figure 3D). Of note, the differences observed between subtypes manifest differently in the cases where the PV neuron models are myelinated or not. For unmyelinated PV neurons, the same field strength leads to less depolarization than is observed in the PC models at the same field strength. However, in the myelinated PV models, the response to the same field strength eventually catches up with the PC models in terms of depolarization. Interestingly, the myelinated PV models also hyperpolarize more aggressively than either the PC or unmyelinated PV models (Figure 3D), suggesting a difference in excitability induced by the applied field. The lack of statistically significant differences observed in any of these poolings suggests limitations to the extent of specificity attainable via NIBS techniques for circuit control. The average *CV*_*V*_ for the pooled subtypes suggests that PC cells are more excitable and experience increased firing. Based on the stronger deviation in this curve compared to the PV models, as well as the similarly shaped curves in the same plot pooled by layers, the majority of the variation within the present selection of models comes from the L4 PC models.

In addition to interrogating the effect of model subtype and/or layer on their contributions to response variability in NIBS, we also verified the impact of neuron orientation within a uniform field (see Supplementary Figure 1). Considering three orientations (0°, 90° and 180°) of an exemplar neuron, in agreement with existing results in transcranial direct current approaches Rahman et al. (2013); Liu et al. (2018); Reato et al. (2019), the neuron's depolarization is influenced by its position particularly in the dendritic and axonal compartments. However, the net response to the applied field remains largely conserved at the soma, and does not differ significantly from the somatic membrane potential polarization observed in the other models (Supplementary Figure 1B). That is, for neurons populating layers 2/3, 4 and 5, uniform electric fields are unable to achieve sufficient specificity of neuron subtypes based on their specific orientation in space.

### Examining the relationship between morphological variability and response specificity

In light of the results shown in Figure 3, it becomes of interest to ask: what drives subtype- and layer-specificity? Specifically, we sought to determine whether any of the characterized morphological attributes substantially influence the response of the neurons to uniform electric fields. Returning to the physical metrics used earlier to describe the neuron subtypes and layers (Figures 1B, 2A–C), the relationship of these properties to response can be investigated by quantifying how morphological characteristics correlate with observed changes in cellular polarization. To examine the trends of individual model responses with respect to applied field strength, we here quantified *susceptibility*, a linear measure of the slope of the response which represents the cell-specific subthreshold somatic polarization per unit electric field applied and is analogous to the polarization length used in prior studies (Radman et al., 2009; Tran et al., 2022). The susceptibility quantifies the predisposition to control by the applied field and can be linked to various morphological traits to assess specificity.

**Figure 4:**
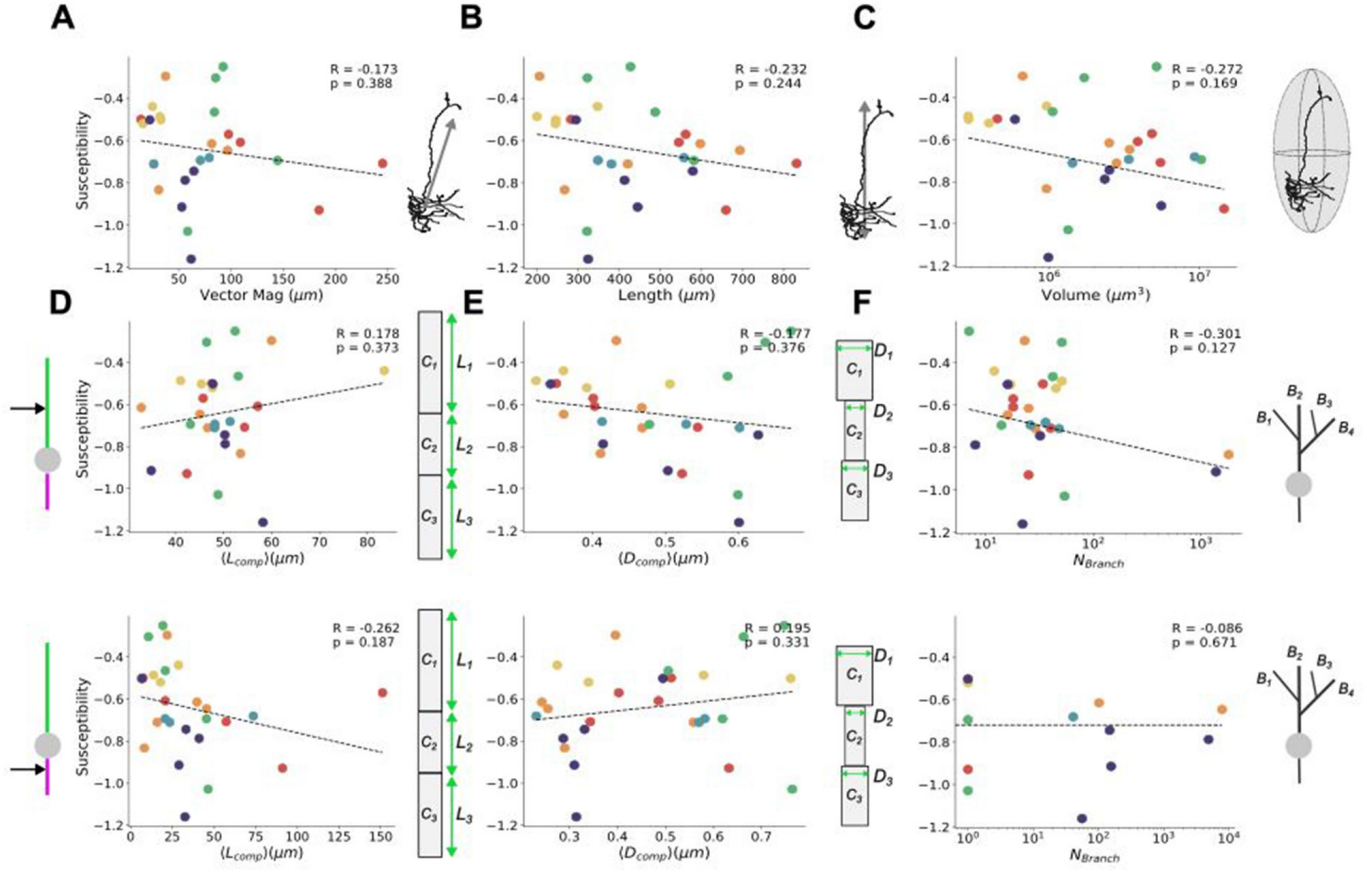
Relating field effects to morphological characterization metrics. Susceptibility of average somatic membrane potential polarization (see Figures 3C, D) per neuron as a function of their **(A)** vector magnitude, **(B)** neuron length, and **(C)** volume occupied. At the level of dendritic (upper) and axonal (lower) compartment properties (see Figure 2), the susceptibility of the average somatic membrane potential polarization is shown as a function of **(D)** average compartment length, **(E)** average compartment diameters, and **(F)** number of branches. All susceptibilities are calculated from response in the upright (0°) orientation. Schematics to the right of each plot correspond to the quantity on their x-axis. The Pearson correlation coefficients, *R*, their significance, *p*, and the line of best fit are found using SciPy's linear regression package (Virtanen et al., 2020). In line with the scheme used in Figures 1, 2, the colors of the points correspond to the layer and cell type of each neuron (red = PC L5, orange = PV L5, yellow = PC L4, green = PV L4, blue = PC L23, and violet = PV L23).

As with the characterization of physical traits, susceptibility can also be broken down into considering both whole-cell and compartmental-scale physical characteristics. Looking at the susceptibility agnostically (irrespective of the neuron subtype or layer) with respect to whole cell metrics (i.e., vector magnitude, length, volume occupied, and the number of branches), all yielded non-significant correlations, while the occupied volume showing the highest correlation (Figure 4). At the compartmental level, investigation of the susceptibility of compartment-level metrics also yielded no significant correlations, with dendritic and axonal branching showing the highest correlation among their respective compartmental attributes. These results suggest that controlling individual cell types with uniform electric fields is limited in its specificity, due to the high variability in cellular morphologies both between cell types and across layers. There was also no notable clustering among the cell types and layers for any of the morphological properties with respect to their susceptibility.

To more rigorously interrogate this result, we re-assessed the relationship between the whole-cell physical metrics (e.g. vector magnitude, volume and length) and susceptibility for partial correlation when holding the relevant covariable morphology traits constant (see Methods). From this more detailed assessment, too, there was no significant partial correlation between these measures and the susceptibility (see Supplementary Figure 2, Supplementary Table 6). Moreover, we then performed dimensionality reduction on all the morphology traits and susceptibilities shown in Figure 4 to create a uniform manifold approximation (UMAP) (see Supplementary Figure 3). From the UMAP, we found no noticeable clustering of any given cell-type and/or layer, suggestive of considerable net morphological similarity across the models.

**Figure 5:**
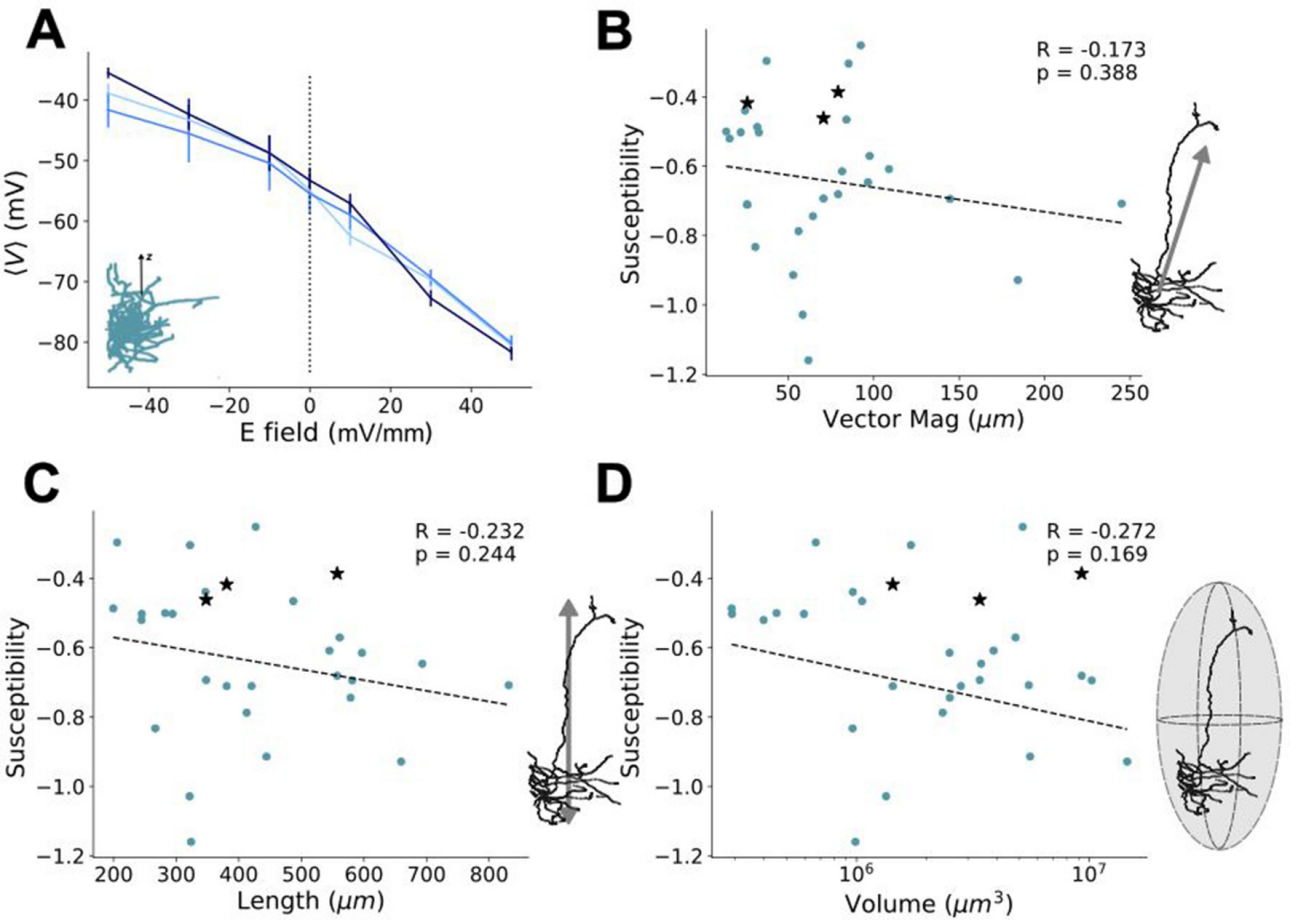
Lack of morphological specificity holds even with fixed ionic properties. **(A)** For a subsample of neuron models (i.e., PC L2/3 models), ionic conductance were set to the same fixed value across all dendritic and axonal compartments (see Methods). Change in polarization to a uniform electric field was measured from the somatic compartment. Different shades of blue denote individual models. Replotting the susceptibility-morphological property scatter plots from Figures 4A–C for **(B)** vector magnitude, **(C)** length and **(D)** volume. The susceptibility of the controlled models from panel **(A)** are added to the scatter plots as black stars.

Based on a range of whole-cell and compartment-based physical metrics, our null results suggest that cellular morphology, on its own, has no significant influence on the specificity of uniform electric fields. However, an important confound remains: the neuron models considered exhibit important compartment-to-compartment variability in biophysical properties (e.g., ion channel conductances) (Technical White Paper: Biophysical Modeling—All Active, 2016; Technical White Paper: Biophysical Modeling—Perisomatic, 2017). Such variability may play an important role in blurring differences in the response of cells across layers and subtypes. To control for this additional source of variability, ionic conductance values of a subset of neuron models were fixed and made equal across all cellular compartments. That is, the ionic conductance values of the L2/3 PC neuron models were extracted from each model and averaged for each ion channel type. The resulting averaged values were then manually applied across each compartment of these models (see Methods). These newly constructed neuron models do not possess any variability in ion channel conductance properties; however, they do retain variability in other compartment-to-compartment level biophysical properties such as resistance, capacitance, and the decay rate of the calcium dynamics. Exposing these biophysically-controlled neuron models to the same uniform E-field protocol as before, our simulations show that morphology does not contribute significantly to specificity: Changes in mean somatic polarization (see Figure 5A) could not be differentiated between the different models. Calculating susceptibility, we also examined the effect of morphological traits. For comparison purposes, we added those to the existing scatter plots computed before (see Figures 5B–D) where biophysical variability was not controlled for. Yet again, we did not find any morphological trait passing the statistical significance threshold, and thus strengthen our null result that no singular morphological trait drives neuron response as independent of biophysical properties.

## Discussion

Previous NIBS studies have demonstrated that single neurons are affected and entrained by low field strengths (Krause et al., 2019; Aberra et al., 2018; Ladenbauer and Obermayer, 2019; Anastassiou et al., 2010; Aspart et al., 2018). The extent of specificity for which NIBS holds over single neurons is less clear. Some experimental studies failed to observe any difference in the entrainment of excitatory and inhibitory neurons (Krause et al., 2022, 2019), yet identifying the source - if any - of NIBS specificity between neurons remains experimentally intractable. Our computational investigation found minimal differences in the acute specificity of neuron subtypes, echoing previous experimental observations (Krause et al., 2022, 2019). No significant correlation between response variability and physical morphology could be identified. These results further align with experimental work revealing that the highly complex, variable morphologies observed in neural tissue had no effect on the conservation of physiological waveforms and circuit functions of neurons (Otopalik et al., 2017b). Similarly, recent computational work in L5 pyramidal cells has shown morphological variance to be insufficient to reproduce electrical variability observed empirically (Arnaudon et al., 2023).

Among the morphological sources of variability we considered was myelination, which is known to influence neuron response to NIBS (Scurfield and Latimer, 2018; Ronzano et al., 2021; Pfeiffer and Benali, 2020). The minor differences in the PV, but not PC, models in the myelinated and unmyelinated versions (see Figure 3D) suggest that response variability due to myelination is non-uniform and, consistent with a recent work (Aberra et al., 2018), requires >15% coverage to manifest. This may help reconcile our observations with previous computational work not considering myelination, which found layer-specific differences in the E-field strength required to depolarize neurons in L2/3 and L5/6 (Radman et al., 2009). However, recent studies involving myelination have shown a minimal difference required to evoke firing from L2/3, L4, and L5 neurons (Aberra et al., 2018, 2020). As the effect of myelination scales with its abundance, less myelinated brain regions, or shorter axons of inhibitory neurons may respond more similarly compared to their unmyelinated equivalents (see Figure 3D), and hence be less depolarized than excitatory neurons. Indeed, such effects may explain differences in our results and other myelinated models that predicted specific activation of cells across subtypes and layers. Where those studies use morphologies from multiple regions of the brain (Komarov et al., 2019), reported differences may hence be attributable to more salient morphological variability present between different brain regions compared to those found within the same region, as we have done in our case. We hypothesize that such region-specific distinctions may also be involved in the discrepancies between those studies and the lack of significant difference found between subtype responses in experimental protocols (Krause et al., 2019).

In addition to layer- and subtype-based neuron groupings, we sought to explore the influence of physical morphology on evoked polarization (see Figure 4). Previous compartment-level studies of simplified neuron models identified multiple physical traits influencing the field strength required to evoke firing (Yi et al., 2017) (while this study considered hippocampal neurons overall, we consider the results of the simplified models comparable). Based on those findings, one could infer that an “optimally susceptible” neuron (that is, one with the lowest required field strength to fire) would have long, thick dendrites with many branches that disperse minimally from the primary axis the applied field lays on. Moreover, such a neuron would have long, narrow axons with minimal branching that disperses widely from the field axis. For the (realistic) models considered here, no class of or individual neuron(s) possess the collective grouping of traits that would require an electric field strength lower than that required by the other neurons to evoke depolarization. In line with this, our simulations did not identify any singular physical trait as the driving source response specificity to the same stimuli between neurons (see Figure 4), even when accounting for partial correlations (see Supplementary Figure 2 and Supplementary Table 6) and the possible influence of variability in compartmental ionic conductances (see Figure 5). These results collectively suggest that the difference between individual neuron models and their response likely results from a convolution of morphological and biophysical properties, rather than morphological traits alone.

Further supporting the seeming convolution of physical traits is the lack of clear clustering observed in the UMAP (Supplementary Figure 3) created from the data in Figure 4, which suggests a significant heterogeneity of and overlap in morphological features and response across the neurons. This aligns with recent work on human neocortical pyramidal cells that, using dimensionality reduction, found considerable intrinsic electrophysiological similarity between layers (Moradi Chameh et al., 2021). Having such overlap in physical traits despite individual morphologies appearing markedly different additionally fits with the ability of cells of widespread morphological variability to create robust firing patterns (Marder et al., 2015; Otopalik et al., 2017a,b, 2019). That is, it supports the idea that neurons may possess widespread degeneracy (Albantakis et al., 2024), whereby they can respond in qualitatively similar ways despite being comprised of different features. Importantly, as is further discussed below in the Limitations section, our null results are constrained in their scope and do not encompass all possible factors that may contribute to neuron-specific responses.

In light of our null results, we feel compelled to mention that we do **not** claim that morphology does not affect neuron response to electric fields, but rather, we conclude that (1) there is no singular trait doing so in isolation; and (2) that the high morphological variability observed across neurons may limit the specificity with which they may be non-invasively targeted. Indeed, our null results suggest that (realistic) morphology is not, in isolation, a defining feature responsible for the observed variability in neuron response to uniform electric field stimulation (see Figure 4). This is in agreement with recent work on detailed biophysical models of L5 PC neurons found morphological variability to be insufficient to reproduce electrical variability (Arnaudon et al., 2023). Moreover, earlier results in somatogastric neurons (Otopalik et al., 2017b), suggest that the electrophysiological properties of neurons can be conserved without complex, morphological diversity. These results support, then, that the approximations made in many models of NIBS protocols that use mean-field type approaches, whether for the whole model population or for subtypes within the population (Huang et al., 2021): The assumptions made in designing such computational models to not include specific morphology in their frameworks may be sufficient for capturing electrical properties.

We believe reporting null results represents a crucial step in evaluating the scope of applicability of NIBS and replicability of existing studies. In recent years, the number of studies pertaining to the use of NIBS has exploded, however, with that momentum, there has been limited replication or consistency in the stimulation protocols used (Hui et al., 2019). Despite their infrequency in publication, null results are often helpful to researchers in furthering research in meaningful directions (de Graaf and Sack, 2018). This is particularly important for the blooming NIBS field, given the extremely vast stimulation parameter space: null results may hence serve to streamline optimizing the framework and scope of NIBS paradigms.

### Limitations

The morphology models used in this work come from a relatively small collection of neurons found in the primary visual cortex of mice. While the small sample size is due to the experimentally limited number of such detailed models available (Technical White Paper: Biophysical Modeling—Perisomatic, 2017), this does contribute to lower statistical power in the results, as is common in the majority of neuroscience studies (Bonapersona et al., 2021). Importantly, the models all being based on mouse primary visual cortex morphologies limit the extrapolation of the results of these simulations to any expectations for human cells, as it has been shown that a number of neuron properties do not scale from non-human to human neurons (Mohan et al., 2015; Beaulieu-Laroche et al., 2021; Chameh et al., 2023). In line with this, recent results showed that human L5 PC neurons have unexpected biophysical properties for their size (Beaulieu-Laroche et al., 2021), despite the composition and allometry of the human cortex scaling relative to other species (Elston et al., 2001; Hattox and Nelson, 2007; Beaulieu-Laroche et al., 2018). Further, as these models are simulated in isolation, they disregard any potential influence from conductive tissue, distance-to-skull or other cell interactions that may bolster response through biophysical mechanisms, such as ephaptic coupling (Reato et al., 2010; Anastassiou et al., 2010; Ladenbauer and Obermayer, 2019; Mahmud and Vassanelli, 2016). White noise is added to account for some of these effects, however, the net efficacy may still be impacted. Additional model-specific limitations can be found in the Allen Institute technical papers and documentation (Technical White Paper: Biophysical Modeling—All Active, 2016; Technical White Paper: Biophysical Modeling—Perisomatic, 2017).

The neuron models were predominantly simulated in one orientation in free space. However, the overall effects of orientation were considered by rotating the neuron models 0°, 90° and 180° about the y-axis and recording from the somatic, axonal and dendritic compartments in all three orientations (see Methods and Supplementary Figure 1). The observed effect of this change is relatively small at the somatic compartment, although the overall polarization of the model changes. NIBS studies of biophysical models found this minimal effect results from the axon branching and myelination, which drastically reduces the effects of orientation on activation threshold (Aberra et al., 2018). The layers considered may also reduce the effect of orientation, as L1 and L6 have previously shown more variability in preferential orientation (Aberra et al., 2020; Liu et al., 2018). The average membrane potential in response to orientation changes is more attenuated at the soma than the dendrites and axons, which are two to three times more susceptible to polarization at their terminals than somas (Rahman et al., 2013).

The models considered in the present work are additionally simulated in isolation, and hence disregard the potential influence of conductive tissue, distance-to-skull, or other cell interactions. One of the immediate possibilities with this framework is that, for real neurons, the minimum field strength required to facilitate depolarization may be greater than in these models where the field is applied uniformly. Alternatively, the synaptic coupling with other neurons may bolster their response and allow for activation at lower field strengths than reported here. To account for this latter possibility, we have added white noise of constant amplitude to mimick recurrent input from other neurons (see Methods, Neuron Models). We acknowledge that this approach neglects the potential effects of network/recurrent correlations, which can bolster response to NIBS far beyond what a single neuron can achieve (Reato et al., 2013; Anastassiou et al., 2010). This is true even in small networks, where ephaptic coupling can influence the strength of field required to entrain cells (Ladenbauer and Obermayer, 2019; Mahmud and Vassanelli, 2016). Therefore, the exact propagation of current through *in vivo* tissue might differ from what is modeled here. This may be of particular importance when considering limits where the neurons are at a greater distance from the current source, or the possibility that connectivity patterns may contribute to the specificity with which neurons can be non-invasively recruited. These important questions are left for future work.

Among factors not considered in the present study are membrane time constants, which vary by multiple orders of magnitude within and between cell types, layers, and brain regions (Moradi Chameh et al., 2021), and reflects cellular excitability and integration of temporally-varying stimuli in the neuron (Fourcaud-Trocmé et al., 2003; Ledoux and Brunel, 2011). This leads to varied responsiveness between neurons to rhythmic input, and contributes to the resultant dynamics of synaptic plasticity in transcranial alternating current stimulation frameworks (Pariz et al., 2023). In addition to synaptic plasticity, by simulating the neurons in isolation, we also neglect potential influences by synaptic inputs, and recurrent connections. This is of particular note, as recurrent connections increase the correlated activity among neurons (Wiechert et al., 2010; Helias et al., 2014) and their absence may have marked effect on neuron response to transcanial electrical stimulation. Moreover, recurrent connectivity influences synaptic low-pass filtering that can introduce slower dynamics in the activity of individual neurons different than those of adaptive currents (Beiran and Ostojic, 2019). Collectively, these factors may influence the response variability of individual neurons, and particularly the selectivity of such neurons in cortical circuit settings.

## Methods

### Neuron models

The models used here were created by the Allen Institute for Brain Science based on the morphologies and biophysical properties of real neurons found in the primary visual cortex of mice (Technical White Paper: Biophysical Modeling—All Active, 2016; Technical White Paper: Biophysical Modeling—Perisomatic, 2017). That is, these computational mathematical models of single neurons were created based on slice electrophysiology and morphology reconstruction data (see below). A primary objective of the Allen Institute in developing these models was to incorporate active dendritic conductances due to their importance in the input-output relationship of cortical neurons (Technical White Paper: Biophysical Modeling—All Active, 2016). This was accomplished by including active, Hodgkin-Huxley nonlinear conductances in the dendrites. For the *excitatory* neuron models there are four separate compartment types incorporated: axonal, somatic, basal dendritic, and apical dendritic. In contrast, the *inhibitory* neuron models only use three types of compartments: axonal, somatic, and dendritic. For both model types active and passive properties are considered.

The morphologies of each neuron are rendered in 3D using the NEURON coding environment from imported SWC files. These models feature a singular, spherical somatic compartment, serving as the initial point from which child branches emerge, extending outward into their respective branches (Technical White Paper: Biophysical Modeling—All Active, 2016; Technical White Paper: Biophysical Modeling—Perisomatic, 2017). As branches are added further from the somatic compartment, their diameters were made to match the expected decrease in diameter observed with increasing branch order. Only models with an overall decrease in dendritic diameter with increased branching order were accepted (Technical White Paper: Biophysical Modeling—All Active, 2016).

Once the morphology of the model is in place, the passive and active properties of each model were fit on electrophysiological data. In the optimization of the 26 free parameters in these models (18 active conductance densities, 4 intracellular *Ca*^2+^ dynamics parameters and four passive parameters), fitting is assessed based on 11 electrophysiological features including time to first spike, time to last spike, as well as action potential width and height (see Technical White Paper: Biophysical Modeling—All Active, 2016 are references therein). For every o those features, an absolute *Z*−score was calculated and the highest score parameter set was retained.

The passive parameters (e.g. specific membrane capacitance, membrane resistance, intracellular resistivity, etc.) are assumed to be uniformly distributed across all compartments and are, for a given model, set to a singular value. The value itself can be found using NEURON's multiple run fitter. As a result, compartment passive parameters were varied to find the best match to experimental voltage traces, under the assumption the active properties were absent (Technical White Paper: Biophysical Modeling—Perisomatic, 2017; Technical White Paper: Electrophysiology, 2017).

In contrast to the passive parameters, the active channel properties are uniformly distributed in space across compartments (Technical White Paper: Biophysical Modeling—All Active, 2016) in the different compartment types (e.g. somatic, axonal, dendritic) such that every type receives an independent set of channels (see Table 1). As with the passive parameters, the numerical values for these active properties were fitted so that the response of the model to step input current matches electrophysiological data. Specifically, the free active parameters include 18 active conductance densities (i.e., *g*_*bar, j*_) which corresponds to the maximum conductance density of type *j* (e.g. Na+) in a given compartment (Technical White Paper: Biophysical Modeling—Perisomatic, 2017). Except for these free active parameters, all other parameters for the active mechanisms are employed based on those from Hay et al. (2011).

**Table 1:** Active properties morphology models.

**Active properties**	**Axon**	**Soma**	**Dendrites**
Ih		✓	✓
Im			✓
Na	✓	✓	✓
Kd	✓		
Kv2-like	✓		
Kv3-like	✓	✓	✓
SK-type potassium	✓	✓	
Low-voltage Ca2+	✓	✓	
High-voltage Ca2+	✓	✓	
Ca2+ decay dynamics	✓	✓	
Ca2+ gamma dynamics	✓	✓	

Reproduced table from Allen Institute technical paper (Technical White Paper: Biophysical Modeling—All Active, 2016) listing all active parameters present in the Biophysical— all active models. Check marks denote the presence of that property in those compartment types.

These free active parameters are complemented by additional parameters that account for intracellular Ca2+ dynamics and Ca2+ entry due to transmembrane currents (Technical White Paper: Biophysical Modeling—All Active, 2016). This model includes a time constant for Ca2+ removal, as well as the binding ratio of the Ca2+ buffer (Technical White Paper: Biophysical Modeling—Perisomatic, 2017), and is given by


(1) $$\begin{array}{l} \frac{dCa_{i}}{dt}=-10000\frac{i_{Ca}\gamma }{2Fd}-\frac{Ca_{i}-m_{Ca_{i}}}{\tau_{d}}, \end{array}$$

where *Ca*_*i*_ is the *Ca*^2+^ concentration at time *t* in compartment *i*, *F* is the Faraday constant, *d* is the depth of the submembrane shell, τ_*d*_ is the removal rate of *Ca*^2+^, γ is the % of free unbuffered *Ca*^2+^, *m*_*Cai*_ is a constant baseline concentration, and *i*_*Cai*_ is the ionic current. With the exception of the calcium dynamics, the active properties of the various ion channels all evolve according to Hodgkin-Huxley equations. The specific equations and parameters for a given model can be found in the “.mod” files downloadable from the Cell Types Database of the Allen Brain Atlas (https://celltypes.brain-map.org/data).

For the purposes of the simulations done in this work, two types of models were considered: those based on pyramidal cell (PC) morphologies and those based on parvalbumin cell (PV) morphologies. A summary of the number of models used from each type and layer is shown in Table 2. All simulations were conducted in a mixed Python—NEURON environment, with the models having been created in a NEURON environment (Hines and Carnevale, 1997; vanRossum, 1995; Technical White Paper: Biophysical Modeling—All Active, 2016). Subsequent data analysis was performed using the NumPy, Matplotlib, Seaborn, SciPy, and Pandas Python libraries (Oliphant, 2006; Hunter, 2007; Waskom, 2021; Virtanen et al., 2020; McKinney et al., 2011).

**Table 2:** Morphological model types.

**Cell type**	**Cortical layer**	**No. morphs**
PC	2/3	3
	4	5
	5	5
PV	2/3	5
	4	4
	5	5

Summary table of the number of morphological models (No. morphs) for each cell type and cortical layer used in the simulations performed here. Pyramidal cells are PC and parvalbumin cells are PV.

In the NEURON environment, the membrane potentials of the models are calculated using the cable equation:


(2) $$\begin{array}{l} \frac{\partial V}{\partial t}+I_{net}=\frac{\partial^{2}V}{\partial x^{2}}, \end{array}$$

where *I*_*net*_ and *V* are the net current (ionic and injected) and membrane potential, respectively. This is then approximated to its spatially discretized form such that the neuron is reduced to a set of connected compartments, and Equation 2 becomes a family of equations,


(3) $$\begin{array}{l} c_{j}\frac{dv_{j}}{dt}+i_{ion,j}=\underset{k}{\sum }\frac{v_{k}-v_{j}}{r_{jk}}+i_{inj,j}, \end{array}$$

where *c*_*j*_ is the membrane capacitance of compartment *j*, *r*_*jk*_ is the resistance between compartment *j* and *k*, *i*_*inj, j*_ are the injected currents, and *i*_*ion, j*_ includes all currents through ionic channel conductances. The right-hand side of the equation is the sum of the axial currents that enter the compartment from its adjacent neighbors. The ionic currents, *i*_*ion*_, of the neuron models then evolve according to *i*_*ion*_ = *g*(*v* − *e*_*ion*_), where *v* is the internal voltage, *e*_*ion*_ is the Nernst potential, and *g* relates the active conductances densities, *g*_*bar*_, to the relevant Hodgkin-Huxley parameters [see individual model “.mod” mechanism files for Equations Technical White Paper: Biophysical Modeling—All Active (2016), and chapters 3 and 4 of the NEURON handbook (Carnevale and Hines, 2006) for detailed expansion on this derivation of the cable equation].

Within this framework, any spatial variation in the membrane current is approximated as its value at the center of a given compartment. Within NEURON's framework, compartments of the same size are grouped together as a section which contains all of these compartments as segments of the section (Hines and Carnevale, 1997; Carnevale and Hines, 2006). This is done for computational efficiency, as Equation 3 may then be re-formulated as,


(4) $$\begin{array}{l} C_{m}\frac{dv_{j}}{dt}+i_{j}=\frac{d}{4R_{a}}\frac{v_{j+1}-2v_{j}+v_{j-1}}{\Delta x^{2}}, \end{array}$$

where Δ*x* and *d* are the compartment length and diameter, respectively. If these are the same between compartments, as is the case for a section, then $R_{a}\Delta x/\pi {\left(d/2\right)}^{2}$ is the axial resistance, and *C*_*m*_π*dΔx* is the compartment capacitance.

The electric field is applied to all compartments in a cell using NEURON's extracellular function (Hines and Carnevale, 1997). We neglect the distance component of the field, that is the field is scaled to be connected in series with the conductance of the last extracellular layer of the model and added in millivolts. For these simulations, we use a uniform electric field with magnitude, *E*, in the positive z-direction, such that each compartment receives a field given by:


(5) $$\begin{array}{l} E_{j}=E\text{cos}\phi +\xi_{j}, \end{array}$$

where *j* is the compartment number, ϕ_*j*_ is the angle between the middle of compartment *j* and the z-axis, and ξ_*j*_ is Gaussian white noise added to that compartment to account for fluctuations and inputs not explicitly modeled, such as synaptic activity or ephaptic interactions, which may modulate neuronal excitability and facilitate activation at lower field strengths. To account for the orientation of the individual compartments, we calculate the angle ϕ_*j*_ based on the *a priori* known Cartesian coordinates of each compartment (Technical White Paper: Biophysical Modeling—All Active, 2016; Hines and Carnevale, 1997). In these calculations, in line with the design of the models detailed previously, the somatic compartment is assumed to be located at the origin.

Taking these steps guarantees that field magnitude in a compartment scales with its orientation (e.g., only compartments in perfect alignment with the field receive its full magnitude). The scaling of the field per-compartment based on compartment orientation is particularly important when we consider the effects of model-orientation on the observed response. When the model is rotated, this is done again using the known Cartesian coordinates of each compartment and a rotation matrix to reorient all compartments to their new positions. The angles, ϕ_*j*_, of the new positions are then calculated and again used to adjust the compartment-wise experienced field strength.

In addition to the applied field, our model also includes background noise ξ, resulting in voltage fluctuations even when *E* = 0*mV*/*mm*. This noise was assumed to be Gaussian white noise (i.e., μ_ξ_ = 0; σ_ξ_ = 4) and was added alongside the field to each compartment. Realizations of this noise are independent across neuron models, compartments and trials. Ten trials each of subthreshold field strengths *E* = {−50, −30, −10, 0, 10, 30, 50}*mV*/*mm* were applied, in line with ranges that have been used for similar models (Yi et al., 2017; Radman et al., 2009). The cell response was read out from the somatic compartment as a membrane potential and the average response across trials was taken to quantify the specificity of the cell.

### Characterizing neural morphology

In the present framework, the Cartesian coordinates for all compartments in the models are known. This allows for both whole-cell and compartmental characterizations of the morphologies to be made. For macroscopic measures, that is characterizations that encompass the whole cell, we take three quantities (1) the vector magnitude, (2) the length in the *z*-direction, and 3) the elliptical volume. We define the vector magnitude as the mean square root of the sum of the squares for the Cartesian coordinates of all compartments in a given model and calculate it to $R_{j}={\langle \sqrt{x^{2}+y^{2}+z^{2}}\rangle }_{N}$, where *N* is the number of compartments in model *j*. For the *z*-direction length (referred to hereafter as length), the value *L* is calculated as simply the difference between the maximum and minimum *z*-direction coordinates [this represents the effective length of the neuron as established in Tran et al. (2022)]. Finally, the elliptical volume is calculated by halving the length (*z*) and equivalent maximum distances in the *x*- and *y*- directions. The ellipsoidal volume is then calculated as:


(6) $$\begin{array}{l} V=\frac{4\pi }{3}\left(\frac{x_{max}}{2}\right)\left(\frac{y_{max}}{2}\right)\left(\frac{z_{max}}{2}\right)=\frac{\pi }{6}(x_{max}y_{max}z_{max}) \end{array}$$

Additionally, at the level of whole cells, we sought to investigate the effects of orientation. To do this, the models were rotated about the *y*-axis using a rotational matrix within the field at θ = 0°, 90°, and 180°. At each orientation, the models were simulated with the same *E* = {−50, −30, −10, 0, 10, 30, 50} mV/mm fields. For these simulations, recordings of the stimulation responses were taken for all compartment types to create a more complete polarization profile. Notably, in these simulations the response is recorded at the center of a given section to approximate the value of all segments within the section.

For the more mesoscopic measures, that is those at the level of the compartments that comprise the models, we consider again three quantities: (1) the average compartmental length, (2) the average compartmental diameter, and (3) the number of branches. For each of these metrics, we further separate into the dendritic and axonal measures. These metrics are defined in the model reconstruction files themselves (Technical White Paper: Biophysical Modeling—Perisomatic, 2017; Hines and Carnevale, 1997), and were subsequently extracted and averaged across morphologies for the same neuron subtype and/or layer.

### Simulations and analysis

For the simulations conducted here, the cells are artificially positioned such that the somatic section, a singular section for all models, is at the origin and all other compartment coordinates are normalized with respect to this compartment. This is done using the *a priori* known Cartesian coordinates for every segment in the models. Additionally, the orientations were taken to be in standard depiction format, that is with the dendritic arbor in the positive z-direction. To improve the biophysical accuracy of the models, myelin was added to the axonal compartments of the neuron models by randomly selecting a fraction of the axonal segments and setting their ionic conductances (and by extension their ionic currents) to be zero. The myelinated segments in a given simulation were chosen from a Bernoulli trial for each axonal segment with a probability of myelination *p* = 0.7 in the PV neuron models and *p* = 0.15 for PC models (see Figure 1C). These values of myelination probability were chosen in line with the percentage of the axon length expected to be myelinated from experimental work on these neuron types (Call and Bergles, 2021).

The physical characteristics of the models were separated based on their layers and subtypes. As the groupings of neurons are small, and therefore cannot be assumed to have normally distributed properties, and each neuron's properties are assumed to be independent of the other neurons, the differences between groupings were assessed for significance using a Mann Whitney U Test from SciPy's statistics library (Virtanen et al., 2020). For the same reasons, comparisons between the responses of neuron groupings were also assessed for significance using Mann Whitney U Tests.

To validate that the observed results are not convolved by the ionic properties, a subset of the neurons were resimulated with their ionic properties manually reset to average values. That is, the conductances, $g_{ion}^{comp}$, of the ionic channels for each of the PC L2/3 models were averaged for each compartment type (somatic, dendritic, apical and axonal). The resultant values, $\langle g_{ion}^{comp}\rangle$, were manually set for each of the corresponding compartments in those models. These models were then re-simulated in the same uniform electric field protocol in the upright (positive *z*−axis) orientation, with their membrane potentials measured again at the somatic compartment.

### Susceptibility

The curves of membrane potential resulting from the application of various applied field strengths as recorded at the somatic compartment in the upright (or 0°) orientation serve as a representation of the susceptibility of a neuron to the applied field. Here, we measure susceptibility, a measure analogous to the polarization length (Radman et al., 2009; Tran et al., 2022), using the slope of the membrane potential polarization curve with respect to applied field strength:


(7) $$\begin{array}{l} S=\frac{{\langle V\rangle }_{-50}-{\langle V\rangle }_{+50}}{\Delta E} \end{array}$$

where Δ*E* is the difference between the E-field strengths for the cases (here equal to -100 *mV*/*mm*) and 〈*V*〉_*i*_ are the average membrane potentials at field strengths *i* = {−50, +50} *mV*/*mm*. As Δ*E* < 0 here, the resulting values of *S* are also negative.

These susceptibility values offer a metric to quantify the influence of the physical morphology characteristics on the response of the neurons to stimulation by looking at their correlations. Correlations of these susceptibilities are calculated with respect to the different morphological traits using SciPy's linear regression function, which calculates the Pearson correlation coefficient, *R*, and its corresponding *p*-value using a Wald Test with t-distribution of the test statistic (Virtanen et al., 2020). These correlations then allow us to discern which morphological trait (if any) may contribute to the specificity of the NIBS protocol. In addition to the linear regression analysis, partial correlations were calculated for morphology traits with their susceptibility using the Pingouin Statistics package in Python (Vallat, 2018). The morphology traits held constant in each of these analyses were decided based on having a significant correlation with the other available ones (see Supplementary Figure 2A).

## Data Availability

Publicly available datasets were analyzed in this study. This data can be found here: https://github.com/AllenInstitute/AllenSDK/tree/master/allensdk/model/biophysical. The repository for the simulations used in this work are available at: https://github.com/djtrotter/morph-var/tree/main.

## Supplementary material

Supplementary Figure 1 Orientation effects on the polarization profile of neurons. **(A)** Average membrane potential per compartment for *E* = −50 mV/mm applied to an example L5 PV neuron model as it is rotated 0°, 90° and 180° (left to right) with respect to the y-axis. Orientation is indicated in upper right of each plot by the arrows on the circles. The lightness of blue on the arrow corresponds to the curves on plots in **(B)**. **(B)** The relationship between the example neuron model orientation and average membrane potential with respect to the applied E-field, error bars are standard deviation. The simplified neuron schematic in the bottom right of each plot indicates which compartment type is being plotted (left to right: somatic, axonal, dendritic). The exact section of the neuron being recorded from is indicated by the color-matched triangles in the rightmost panel of **(A)**. In the left panel of **(B)** the average membrane potential at 0° orientation from the somatic compartment of all models is indicated by the gray line with the error bars as their standard deviation. The inter-model mean was compared with the single model mean using a Mann Whitney U Test and no significant difference was found (*p*>0.05).

Supplementary Figure 2 Partial correlation analysis shows no significant correlation between susceptibility and morphology traits. **(A)** Heat map of the spearman correlation coefficient between each of the morphology traits considered in this work. Significance (*p*-values) for these correlations are written on each block rounded to two decimal places. Cases with *p* < 0.01 (shown as *p* = 0.0 in heatmap) are considered correlated and controlled for in the partial correlation analysis. The exact values for the correlation coefficients and *p*-values are reported in Supplemental Tables 4 and 5. **(B)** Heatmap of the spearman partial correlation coefficients between the susceptibility and each morphology trait when controlling for significantly correlated traits as determined from **(A)**. The *p*-values are shown on each block. The exact values for the correlation coefficients and *p* values are reported in Supplemental Table 6.

Supplementary Figure 3 Dimensionality reduction shows no clustering between neuron types or layers Performing dimensionality reduction on the nine morphology traits considered in Figure 4 and susceptibilities of the neurons to create a uniform manifold approximation (UMAP) shows no clear clustering of the neurons in reduced space.
