# Supplementary material for: Morphological variability may limit single-cell specificity to electric field stimulation
Source: Front Synaptic Neurosci. 2025 Aug 5;17:1621352. doi: 10.3389/fnsyn.2025.1621352 (PMC12361131; doi:10.3389/fnsyn.2025.1621352)
Supplement: Supplementary file 1 [file Supplementary_file_1.pdf]

Cell type	Layer	Mean Vec. mag.	Stdv Vec. Mag.	Mean Len.	Stdv Len.	Mean Vol.	Stdv Vol.
**PC**	2/3	58.648	23.291	428.531	91.967	4.69e6	3.33e6
	4	92.996	28.292	427.732	99.558	3.90e6	3.49e6
	5	129.959	78.986	575.823	178.655	5.83e6	4.68e6
**PV**	2/3	51.509	15.179	410.651	100.481	2.40e6	1.75e6
	4	26.325	6.868	258.925	54.022	4.85e5	2.79e5
	5	54.469	28.911	436.551	186.563	2.07e6	1.07e6

Table 1. Mean and standard deviation of the vector magnitudes, Vec. mag., lengths, Len., and Volumes, Vol., for each cell type (PC or PV) and cortical layer (Layer) shown in Fig 1B. Stdv is the standard deviation

Cell type	Layer	Mean Len.	Stdv Len.	Mean Diam.	Stdv Diam.	Mean $N_{Branches}$	Stdv $N_{Branches}$
**PC**	2/3	49.199	1.514	0.514	0.077	36.66	8.99
	4	48.749	3.723	0.594	0.065	33.6	19.39
	5	49.410	5.498	0.444	0.075	27.0	8.763
**PV**	2/3	48.270	7.564	0.498	0.108	291.2	543.45
	4	54.389	16.982	0.396	0.068	31.25	16.97
	5	47.602	9.101	0.428	0.040	380.4	714.31

Table 2. Mean and standard deviation of the dendritic lengths, Len., diameters, Diam., and branches, $N_{Branches}$, for each cell type (PC or PV) and cortical layer (Layer) shown in the top row of Fig 2. Stdv is the standard deviation

Cell type	Layer	Mean Len.	Stdv Len.	Mean Diam.	Stdv Diam.	Mean $N_{Branches}$	Stdv $N_{Branches}$
**PC**	2/3	39.25	24.31	0.461	0.163	13.66	19.3
	4	28.57	14.63	0.659	0.4	0.48	19.39
	5	65.55	51.86	0.475	0.098	0.2	0.4
**PV**	2/3	28.55	11.56	0.347	0.075	1045.8	1911.98
	4	16.63	8.135	0.488	0.194	0.25	0.43
	5	26.28	14.18	0.348	0.118	1586.6	3122.4

Table 3. Mean and standard deviation of the axonal lengths, Len., diameters, Diam., and branches, $N_{Branches}$, for each cell type (PC or PV) and cortical layer (Layer) shown in the bottom row of Fig 2. Stdv is the standard deviation

Trait	Len	Vol	Vec Mag	D Len	D Bran	D Diam	A Len	A Bran	A Diam
**Len**	1.00000	0.85409	0.76495	-0.15873	-0.24312	0.23931	0.72283	0.36273	-0.25274
**Vol**	0.85409	1.00000	0.76129	-0.14469	-0.16891	0.32356	0.67827	0.27255	-0.06410
**Vec Mag**	0.76495	0.76129	1.00000	-0.06227	-0.21258	0.38705	0.61782	0.11441	0.08852
**D Len**	- 0.15873	-0.14469	-0.06227	1.00000	-0.12523	0.13736	-0.01831	-0.27726	-0.21978
**D Bran**	-0.24312	-0.16891	-0.21258	-0.12523	1.00000	0.16731	-0.13012	-0.12659	0.06291
**D Diam**	0.23931	0.32356	0.38705	0.13736	0.16731	1.00000	0.16056	-0.01749	0.39987
**A Len**	0.72283	0.67827	0.61782	-0.01831	-0.13012	0.16056	1.00000	0.48319	-0.31562
**A Bran**	0.36273	0.27255	0.11441	-0.27726	-0.12659	-0.01749	0.48319	1.00000	-0.49329
**A Diam**	-0.25274	-0.06410	0.08852	-0.21978	0.06291	0.39987	-0.31562	-0.49329	1.00000

Table 4. Spearman correlations of the morphology traits shown in Supplemental Fig 2A.

Trait	Len	Vol	Vec Mag	D Len	D Bran	D Diam	A Len	A Bran	A Diam
**Len**	0.00E+0	1.45E-8	3.37E-6	4.29E-1	2.21E-1	2.29E-1	2.05E-5	6.29E-2	2.03E-1
**Vol**	1.46E-8	0.00E+0	4.00E-6	4.71E-1	3.99E-1	9.97E-2	1.01E-4	1.69E-1	7.51E-1
**Vec Mag**	3.37E-6	4.00E-6	0.00E+0	7.57E-1	2.87E-1	4.69E-2	5.94E-4	5.69E-1	6.61E-1
**D Len**	4.29E-1	4.71E-1	7.57E-1	0.00E+0	5.33E-1	4.94E-1	9.28E-1	1.64E-1	2.71E-1
**D Bran**	2.21E-1	3.99E-1	2.87E-1	5.33E-1	0.00E+0	4.15E-1	5.18E-1	5.29E-1	7.55E-1
**D Diam**	2.29E-1	9.97E-2	4.69E-2	4.94E-1	4.15E-1	0.00E+0	4.24E-1	9.31E-1	3.87E-2
**A Len**	2.05E-5	1.01E-4	5.94E-4	9.28E-1	5.18E-1	4.24E-1	0.00E+0	1.07E-2	1.09E-1
**A Bran**	6.29E-2	1.69E-1	5.69E-1	1.64E-1	5.29E-1	9.31E-1	1.07E-2	0.00E+0	8.93E-3
**A Diam**	2.03E-1	7.51E-1	6.61E-1	2.71E-1	7.55E-1	3.87E-2	1.09E-1	8.93E-3	0.00E+0

Table 5. Significance (p-values) of Spearman correlations of the morphology traits shown in Supplemental Fig 2A and Table 4.

Morphology trait	r	p
Length	0.03413	0.87419
Volume	-0.24106	0.25648
Vector Mag	0.33154	0.11349
Dend Length	0.17849	0.37305
Dend Branches	-0.30075	0.12741
Dend Diameter	-0.17740	0.37602
Ax Length	-0.39228	0.05795
Ax Branches	-0.08573	0.67069
Ax Diameter	0.19463	0.33064

Table 6. The numerical values of the spearman partial correlation coefficients, r, and their significance values, p, for the morphology traits and susceptibilities as plotted in Supplemental Fig 2B.
