# Supplementary figures and images for: Morphological variability may limit single-cell specificity to electric field stimulation
Source: Front Synaptic Neurosci. 2025 Aug 5;17:1621352. doi: 10.3389/fnsyn.2025.1621352 (PMC12361131; doi:10.3389/fnsyn.2025.1621352)

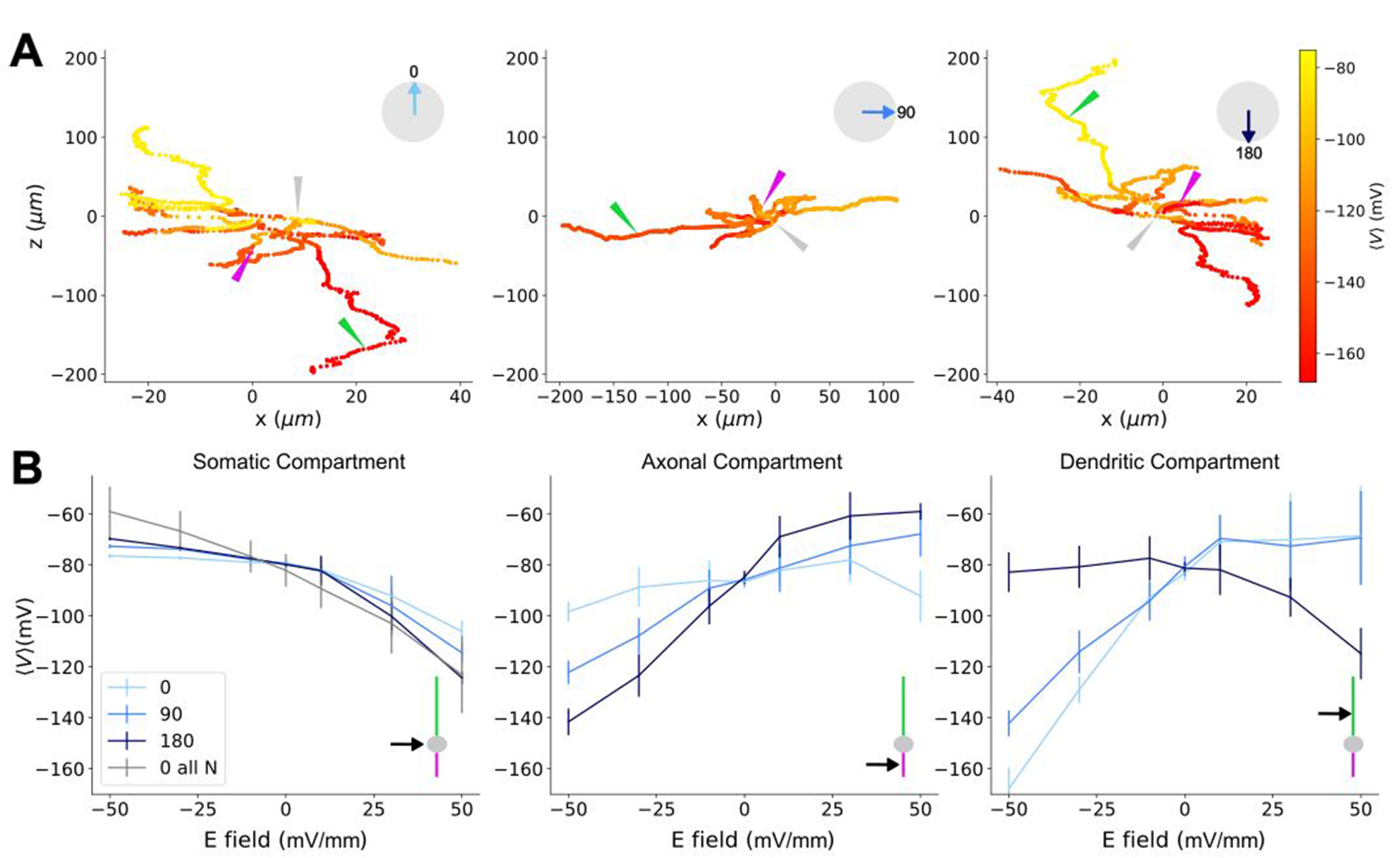

Supplement: Supplementary Figure 1 — Orientation effects on the polarization profile of neurons. (A) Average membrane potential per compartment for E = −50 mV/mm applied to an example L5 PV neuron model as it is rotated 0°, 90° and 180° (left to right) with respect to the y-axis. Orientation is indicated in upper right of each plot by the arrows on the circles. The lightness of blue on the arrow corresponds to the curves on plots in (B). (B) The relationship between the example neuron model orientation and average membrane potential with respect to the applied E-field, error bars are standard deviation. The simplified neuron schematic in the bottom right of each plot indicates which compartment type is being plotted (left to right: somatic, axonal, dendritic). The exact section of the neuron being recorded from is indicated by the color-matched triangles in the rightmost panel of (A). In the left panel of (B) the average membrane potential at 0° orientation from the somatic compartment of all models is indicated by the gray line with the error bars as their standard deviation. The inter-model mean was compared with the single model mean using a Mann Whitney U Test and no significant difference was found (p>0.05). [file Image_1.jpg]

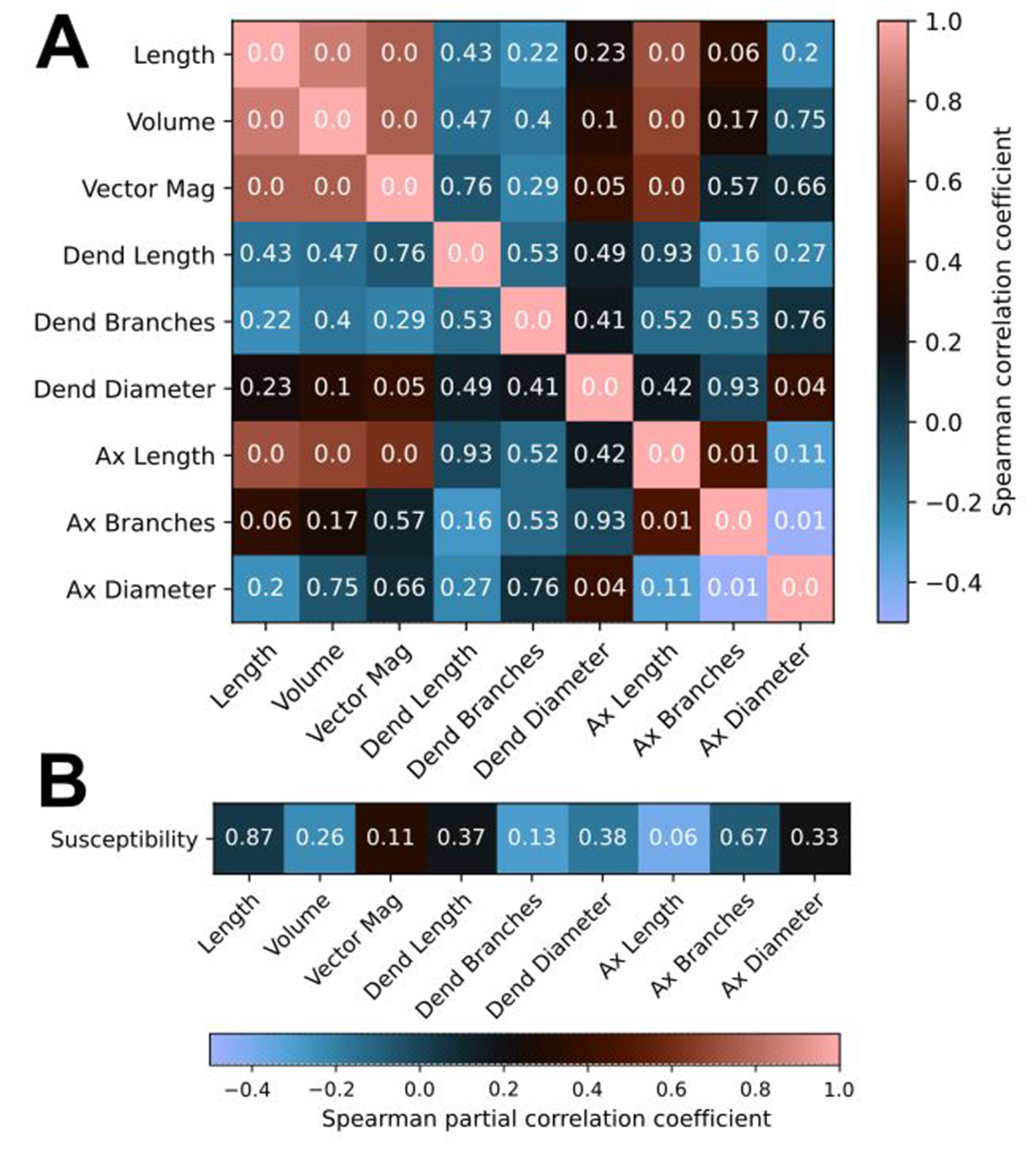

Supplement: Supplementary Figure 2 — Partial correlation analysis shows no significant correlation between susceptibility and morphology traits. (A) Heat map of the spearman correlation coefficient between each of the morphology traits considered in this work. Significance (p-values) for these correlations are written on each block rounded to two decimal places. Cases with p < 0.01 (shown as p = 0.0 in heatmap) are considered correlated and controlled for in the partial correlation analysis. The exact values for the correlation coefficients and p-values are reported in Supplemental Tables 4 and 5. (B) Heatmap of the spearman partial correlation coefficients between the susceptibility and each morphology trait when controlling for significantly correlated traits as determined from (A). The p-values are shown on each block. The exact values for the correlation coefficients and p values are reported in Supplemental Table 6. [file Image_2.jpg]

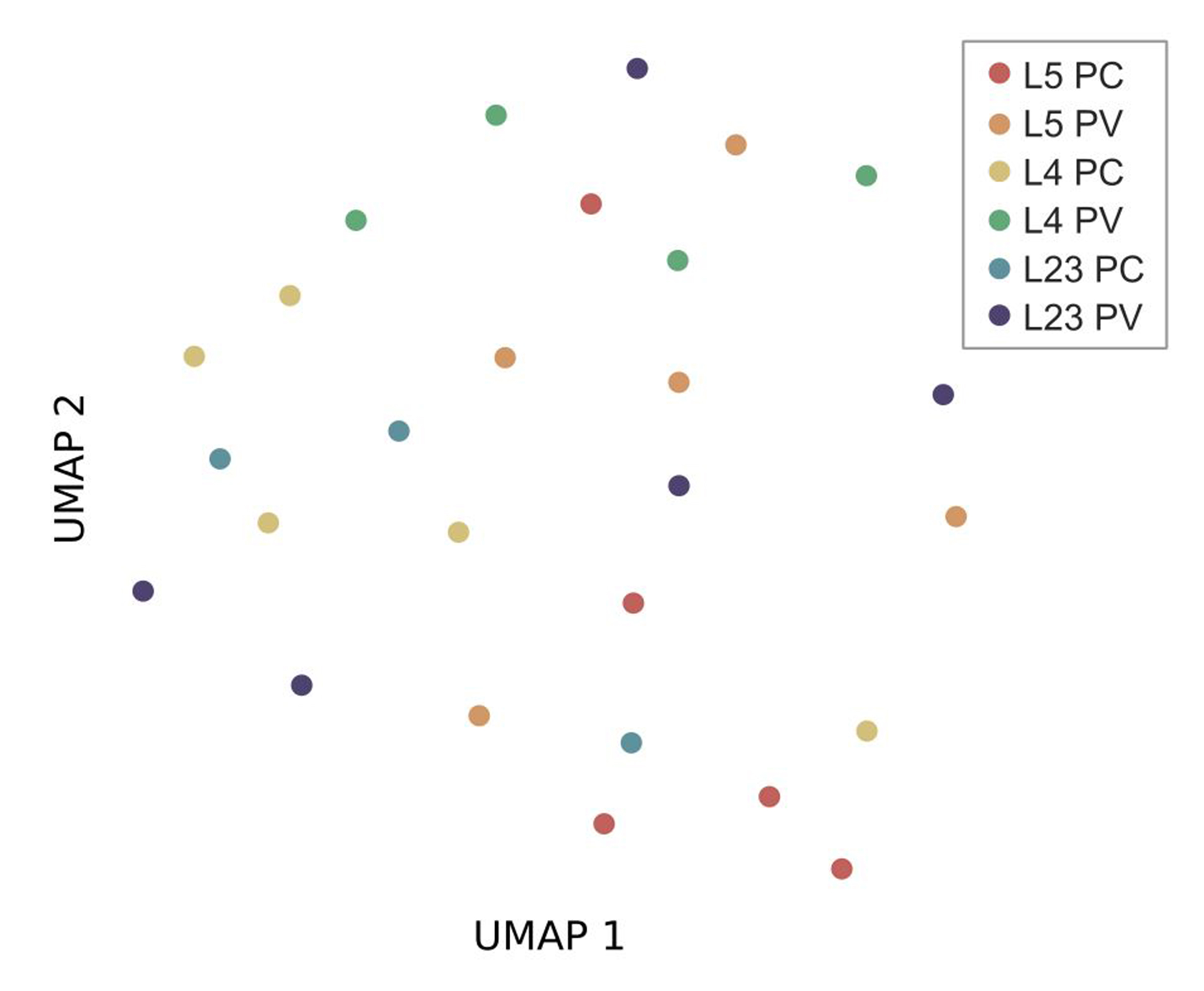

Supplement: Supplementary Figure 3 — Dimensionality reduction shows no clustering between neuron types or layers Performing dimensionality reduction on the nine morphology traits considered in Figure 4 and susceptibilities of the neurons to create a uniform manifold approximation (UMAP) shows no clear clustering of the neurons in reduced space. [file Image_3.jpg]
